# Development of dynamic nomogram for predicting cancer-specific survival in hepatoid adenocarcinoma: A comprehensive SEER-based population analysis

**DOI:** 10.17305/bb.2024.10445

**Published:** 2024-10-01

**Authors:** Qing-Zhe Wang, Yi-Xin Zhou, Xiao-Li Mu, Jia-Ling Wang, Shuang Zhang, Ye Chen

**Affiliations:** 1 Department of Targeting Therapy and Immunology, Cancer Centre, West China Hospital, Sichuan University, Chengdu, China; 2 Division of Abdominal Tumor Multimodality Treatment, Department of Radiation Oncology, Cancer Center, West China Hospital, Sichuan University, Chengdu, China

**Keywords:** Hepatoid adenocarcinoma (HAC), cancer-specific survival (CSS), Surveillance, Epidemiology, and End Results (SEER) database, nomogram

## Abstract

Hepatoid adenocarcinoma (HAC) is a poorly differentiated extrahepatic tumor that can produce alpha-fetoprotein (AFP). The literature does not provide a comprehensive understanding of the prognostic factors for HAC. Therefore, we present a novel nomogram to predict the cancer-specific survival (CSS) of patients with HAC. We analyzed 265 cases of HAC from the Surveillance, Epidemiology, and End Results (SEER) database spanning from 2004 to 2015. Using a Cox proportional hazard regression model, we identified several risk factors and incorporated them into our predictive nomogram. The nomogram’s predictive ability was assessed by utilizing the concordance index (C-index), calibration curve, and receiver operating characteristic (ROC). Results from a multivariate Cox regression showed that CSS was independently correlated with liver metastasis, surgery, and chemotherapy. Our nomogram had a C-index of 0.71 (95% CI 0.71–0.96). Furthermore, calibration curves demonstrated concordance between the predicted survival probability from the nomogram and the observed survival probability. The areas under the curve (AUCs) for 6-month, 1-, and 3-year survival were 0.80, 0.82, and 0.88, respectively. Our study successfully formulated a prognostic nomogram that offers promising predictions for the 6-month, 1-, and 3-year CSS of patients with HAC. This nomogram holds potential for practical use in guiding treatment decisions and designing clinical trials.

## Introduction

Hepatoid adenocarcinoma (HAC) is identified as a type of extrahepatic adenocarcinoma that shares similar morphological characteristics with primary hepatocellular carcinoma (HCC). HAC has been primarily reported in the gastrointestinal tract, especially in the stomach, accounting for 0.39%–1.6% of all gastric cancers [1, 2]. It can also be discovered in the gall bladder, lung, ovary, bladder, and rectum [3, 4]. Patients with HAC are commonly correlated with elevated serum alpha-fetoprotein (AFP) and early metastases of the lymph node, lung, and liver [1, 5, 6]. HAC and HCC share many similar clinicopathological features, including elevated serum AFP, hepatoid morphology (resembling hepatocytes), and positive immunoreactivity with AFP and carcinoembryonic antigen (CEA) [7]. In clinical management, it is necessary to distinguish HAC from HCC or other conventional carcinomas due to their higher metastasis rate and lower survival rate.

Previous research has demonstrated surgical resection as the primary treatment for HAC, whereas recurrence still may happen after R0 resection [8]. Generally, the current American Joint Committee on Cancer (AJCC) staging systems for various cancers were recognized as standard evaluating systems for predicting patients’ survival. However, current clinical studies of prognostic factors often refer to the conventional cancers of the corresponding organs, regardless of the specificity of HAC. So far, the research based on prognostic factors of HAC is restricted to HAC in the stomach (HAS) and quite limited [9]. Therefore, we aimed to analyze the prognostic factors of HAC patients based on the Surveillance, Epidemiology, and End Results (SEER) database and constructed a prognostic nomogram based on these factors.

## Materials and methods

### Patient selection

**Figure 1.**
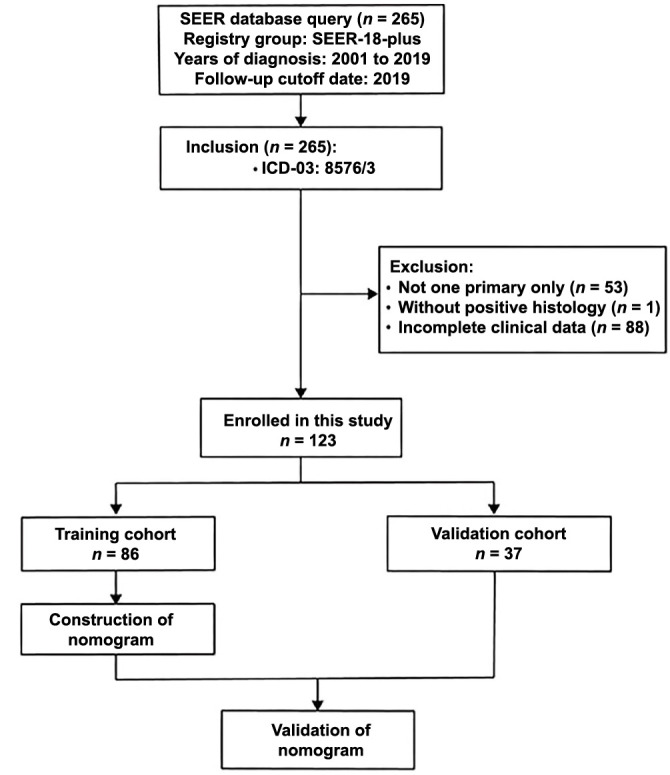
**The flow diagram of our study.** SEER: Surveillance, Epidemiology, and End Results.

Between 2004 and 2015, we retrieved data on 265 initial cases of HAC from the SEER database. Individuals lacking clinicopathologic features were not included in the analysis. Figure 1 displays the specific criteria and data selection procedure in detail. The International Classification of Diseases for Oncology, Third Revision, and Histological Type Codes of 8576/3 were utilized to determine the inclusion criteria. Data extracted from each patient included age, sex, race, year of diagnosis, marital status, AJCC 6th edition T, N, and M stages, histologic type, surgery, radiotherapy, chemotherapy, vital status, survival months, and causes of death. Metastasized locations included the brain, liver, lung, and bone. The final study group consisted of 123 cases with a HAC diagnosis. To develop and validate nomograms, we randomly assigned 37 patients to the validation group and 86 patients to the training cohort from the SEER database.

### Endpoints’ definition

As the length of time from the date of diagnosis to the date of death is specifically related to cancer, cancer-specific survival (CSS) was defined as the primary outcome. Overall survival (OS), the secondary outcome, was defined as the amount of time that has passed between the last follow-up or the date of death for any reason.

### Ethical statement

As the SEER database is publicly accessible, no ethical committee review or approval was required for using this data.

### Statistical analysis

SEER*Stat software version 8.3.9.0 was used to extract patient data. Categorical variables were expressed as percentages. Patients were randomly assigned to the training and validation cohort at a ratio of 7:3. The two groups’ baseline characteristics were compared using the chi-squared test. Cumulative survival curves were generated using the Kaplan–Meier method and compared using the log-rank test. Univariate and multivariate survival analyses were conducted using the Cox proportional hazards model. Hazard ratios (HRs) and 95% confidence intervals (CIs) were established. Multivariate Cox analysis included significant variables (*P* < 0.05 in univariate analysis, *P* < 0.1 in Kaplan–Meier, or clinically noteworthy). Based on factors with *P* < 0.05 in the multivariate analysis, the nomogram was created. Predictive power has been evaluated using the C-index, ROC curve, and calibration curve (1000 bootstrap resamples). Better prediction can be seen by a higher C-index. A curve of calibration with a slope of 1 (the gray line) was utilized to compare the actual results against the projected results. The ROC curve’s area under the curve (AUC) value indicates model discrimination. The effects of the model on clinical net benefit under various thresholds were examined using decision analysis and clinical impact curves. Patients were divided into high-risk and low-risk groups based on the cut-off risk value, which was established by calculating the median risk score for every patient in the training and validation cohorts.

R 4.1.2 software (http://www.rproject.org) and the IBM SPSS 22.0 program (IBM Corporation, Armonk, New York) were used for statistical analysis. R 4.1.2 and shinyapps.io were used in the construction and validation of the nomogram. The dynamic nomogram was constructed using the R package “DynNom.” *P* < 0.05 was regarded as statistically significant in all two-sided statistical tests.

## Results

### Baseline characteristics

After excluding certain patients, a total of 123 individuals were included in the trial, with 86 in the training group and 37 in the validation group (Figure 1). The baseline characteristics of all enrolled patients are summarized in Table 1. In the training cohort, the 6-month, 1- and 3-year CSS rate was 52.8%, 39.8%, and 23%, respectively. In the validation cohort, the 6-month, 1-, and 3-year CSS rates were 45%, 36%, and 22.9%, respectively. The median CSS for training and validation groups was seven and five months, and the median OS was five and five months, respectively. In both cohorts, most patients were white, male, and middle-aged or elderly. The distribution of clinical stages for both groups was generally T3-4, N0, and M1. A total of 57% and 54% of patients in the training and validation group, respectively, had distant metastasis, including bone (12.8%; 13.5%), brain (12.8%; 13.5%) liver (12.8%; 10.8%), and lung (16.3%; 16.2%). The majority of patients were with advanced clinical grades. Most patients underwent surgery, with over half receiving chemotherapy, and less than 40% receiving radiotherapy. All variables were comparable between the two groups.

**Table 1:** Clinicopathological features and treatment background of all patients in both the training and validation cohorts at baseline

**Characteristics**	**Training cohort *N* ═ 86, *n* (%)**	**Validation cohort *N* ═ 37, *n* (%)**	***P* value**
*Age (years)*			
<58	14 (16.3)	6 (16.2)	0.612
58–65	27 (31.4)	11 (29.7)	
66–74	19 (22.1)	12 (32.4)	
≥74	26 (30.2)	8 (21.6)	
*Sex*			
Male	51 (59.3)	25 (67.6)	0.507
Female	35 (40.7)	12 (32.4)	
*Race*			
White	67 (77.9)	29 (78.4)	0.991
Black	9 (10.5)	4 (10.8)	
Other	10 (11.6)	4 (10.8)	
*Marital status*			
Married	49 (57)	14 (37.8)	0.071
Unmarried	32 (37.2)	22 (59.5)	
Unknown	5 (5.8)	1 (2.7)	
*Site*			
Lung	36 (41.9)	15 (40.5)	1.000
Other	50 (58.1)	22 (59.5)	
*T classification*			
T0	2 (2.3)	0 (0.0)	0.434
T1-2	27 (31.4)	15 (40.5)	
T3-4	40 (46.5)	18 (48.6)	
Tx	17 (19.8)	4 (10.8)	
*N classification*			
N0	37 (43.0)	19 (51.4)	0.560
N1-3	31 (36.0)	13 (35.1)	
Nx	18 (20.9)	5 (13.5)	
*M classification*			
M0	34 (39.5)	14 (37.8)	0.551
M1	49 (57.0)	20 (54.1)	
Mx	3 (3.5)	3 (8.1)	
*Bone metastasis*			
No	42 (48.8)	17 (45.9)	0.635
Yes	11 (12.8)	3 (8.1)	
Unknown	33 (28.4)	17 (45.9)	
*Brain metastasis*			
No	46 (53.5)	15 (40.5)	0.367
Yes	11 (12.8)	5 (13.5)	
Unknown	33 (38.4)	17 (45.9)	
*Lung metastasis*			
No	41 (47.7)	16 (43.2)	0.799
Yes	11 (12.8)	4 (10.8)	
Unknown	34 (39.5)	17 (45.9)	
*Liver metastasis*			
No	40 (46.5)	14 (37.8)	0.625
Yes	14 (16.3)	6 (16.2)	
Unknown	32 (37.2)	17 (45.9)	
*Grade*			
1-2	4 (4.7)	2 (5.4)	0.583
3-4	27 (31.4)	15 (40.5)	
Unknown	55 (64.0)	18 (48.6)	
*Surgery*			
Yes	65 (75.6)	25 (67.6)	0.485
No/unknown	21 (24.4)	12 (32.4)	
*Radiation*			
Yes	29 (33.7)	14 (37.8)	0.816
No/unknown	57 (66.3)	23 (62.2)	
*Chemotherapy*			
Yes	37 (43.0)	18 (48.6)	0.706
No/unknown	49 (57.0)	19 (51.4)	

### Factor prediction using univariate and multivariate analyses

**Figure 2.**
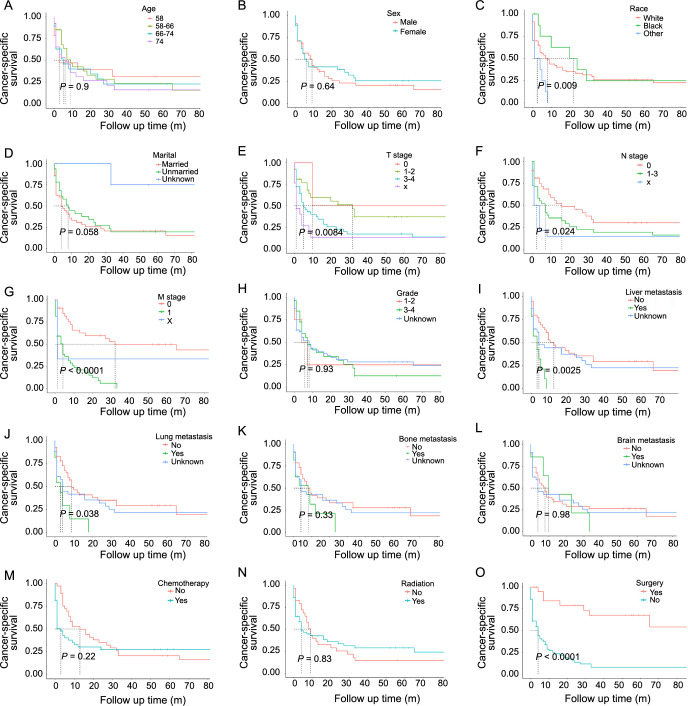
**Kaplan–Meier curves of CSS based on (A) age, (B) sex, (C) race, (D) marital, (E) T stage, (F) N stage, (G) M stage, (H) grade, (I) liver metastasis, (J) lung metastasis, (K) bone metastasis, (L) brain metastasis, (M) chemotherapy, (N) radiation, and (O) surgery.** CSS: Cancer-specific survival.

The training cohort’s CSS was predicted by each variable using the Cox proportional hazards model (Table 2). Univariate Cox analysis indicated that races other than white or black, T3-4 stage, N1-3 stage, M1 stage, liver metastasis, and no surgery were significantly correlated with worse CSS. The Kaplan–Meier curve and log-rank analysis were also applied as a complementation to recognize possible prognostic factors, which showed that other races, unmarried, advanced T, N, M stages, liver, lung metastasis, and no surgery were significantly correlated with poorer prognosis (Figure 2). According to the results, race, marital, TNM classification, surgery, and chemotherapy were then included in the multivariate analysis. Due to the multi-collinearity bias between the M stage and liver/lung metastasis, only the M stage was included in the multivariate analysis. Chemotherapy was also included out of precious research and clinical consideration [10]. The multivariate analysis showed that M stage, surgery, and chemotherapy were independent prognostic factors for patients with HAC.

**Table 2:** Univariate and multivariate Cox analyses of the capacity of each factor to predict CSS

**Characteristics**	**Univariate**			**Multivariate**		
	**HR**	**95% CI**	***P* value**	**HR**	**95% CI**	***P* value**
*Age (vs<58)*						
58–65	3.084	1.060–8.973				
66–74	4.072	1.401–11.838	0.078			
≥74	2.618	0.814–8.424				
*Sex*						
Female vs Male	0.729	0.349–1.523	0.400			
*Race*						
Black vs White	0.468	0.141–1.554	0.008*	0.393	0.141–1.100	0.253
Other vs White	3.827	1.445–10.138		1.217	0.446–3.315	
*Grade*						
II vs I	0.751	0.257–2.199				
III vs I	0.863	0.357–2.087	0.959			
IV vs I	0.856	0.349–2.098				
*Marital status*						
Unmarried vs Married	1.025	0.516–2.039	0.508	0.870	0.475–1.594	0.249
Unknown vs Married	0.433	0.098–1.905		0.173	0.022–1.392	
*Site*						
Others vs Lung	0.681	0.317–1.461	0.324			
*T classification*						
T1-2 vs T0	1.457	0.192–11.051		2.381	0.295–19.229	
T3-4 vs T0	2.771	0.378–20.339	0.020*	3.127	0.446–3.315	0.726
Tx vs T0	4.544	0.592–34.860		2.732	0.308–24.249	
*N classification*						
N1-3 vs N0	1.530	2.656–3.896	0.040*	1.085	0.583–2.020	0.643
Nx vs N0	2.406	1.214–4.767		0.924	0.354–2.411	
*M classification*						
M1 vs M0	4.267	2.335–7.800	<0.001*	2.368	1.083–5.175	0.039*
Mx vs M0	1.940	0.446–8.441		0.739	0.119–4.590	
*Bone metastasis*						
Yes vs No	1.719	0.805–3.670	0.375			
Unknown vs No	1.133	0.658–1.951				
*Brain metastasis*						
Yes vs No	1.078	0.420–2.764	0.984			
Unknown vs No	1.033	0.609–1.752				
*Lung metastasis*						
Yes vs No	2.513	1.159–5.448	0.065			
Unknown vs No	1.272	0.740–2.189				
*Liver metastasis*						
Yes vs No	3.112	1.509–6.420	0.008*			
Unknown vs No	1.240	0.709–2.169				
*Grade*						
III-IV vs I-II	0.985	0.294–3.322	0.937			
Unknown vs I-II	0.862	0.278–2.924				
*Radiation*						
Yes vs No	0.948	0.567–1.583	0.838			
*Surgery*						
Yes vs No	0.174	0.078–0.388	<0.001*	0.251	0.090–0.698	0.008*
*Chemotherapy*						
Yes vs No	0.741	0.448–1.227	0.244	0.505	0.279–0.914	0.024*

Bold values with asterisks indicate statistical significance. CSS: Cancer-specific survival; HR: Hazard ratio.

### Constructing and verifying the nomogram

**Figure 3.**
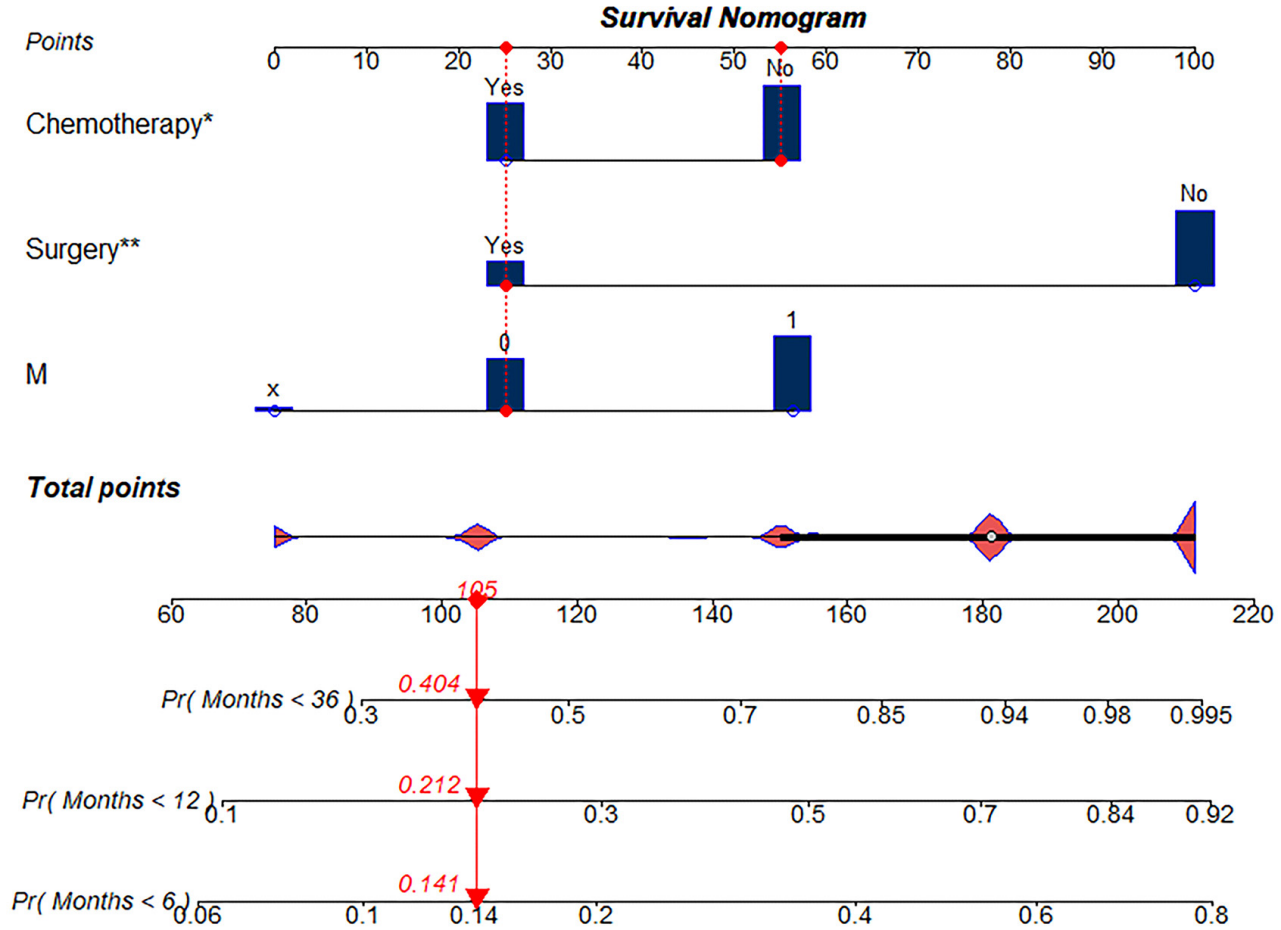
**Nomogram for predicting survival probabilities of 6-month, 1-, and 3-year CSS of patients with HAC.** ^*^P < 0.05, ^**^P < 0.01. CSS: Cancer-specific survival; HAC: Hepatoid adenocarcinoma.

**Figure 4.**
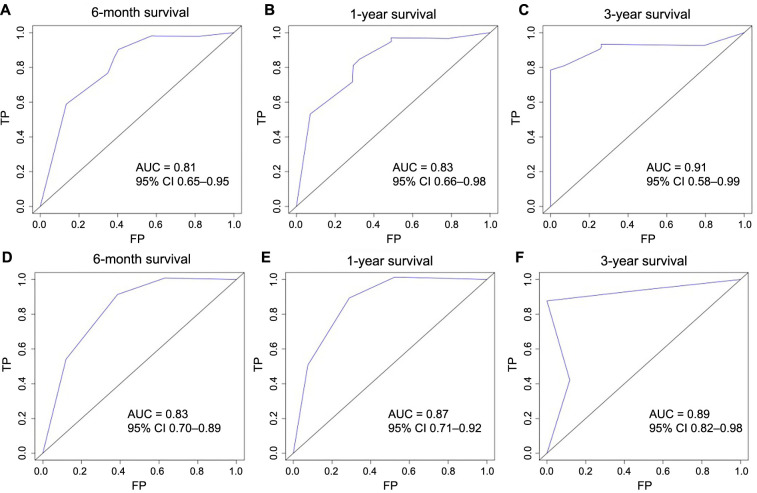
**The ROC curves of the nomogram for prognosis prediction of the 6-month, 1-, and 3-year CSS in the training (A–C) and validation (D–F) groups.** ROC: Receiver operating characteristic; CSS: Cancer-specific survival; TP: True positive; FP: False positive; AUC: Area under the curve.

**Figure 5.**
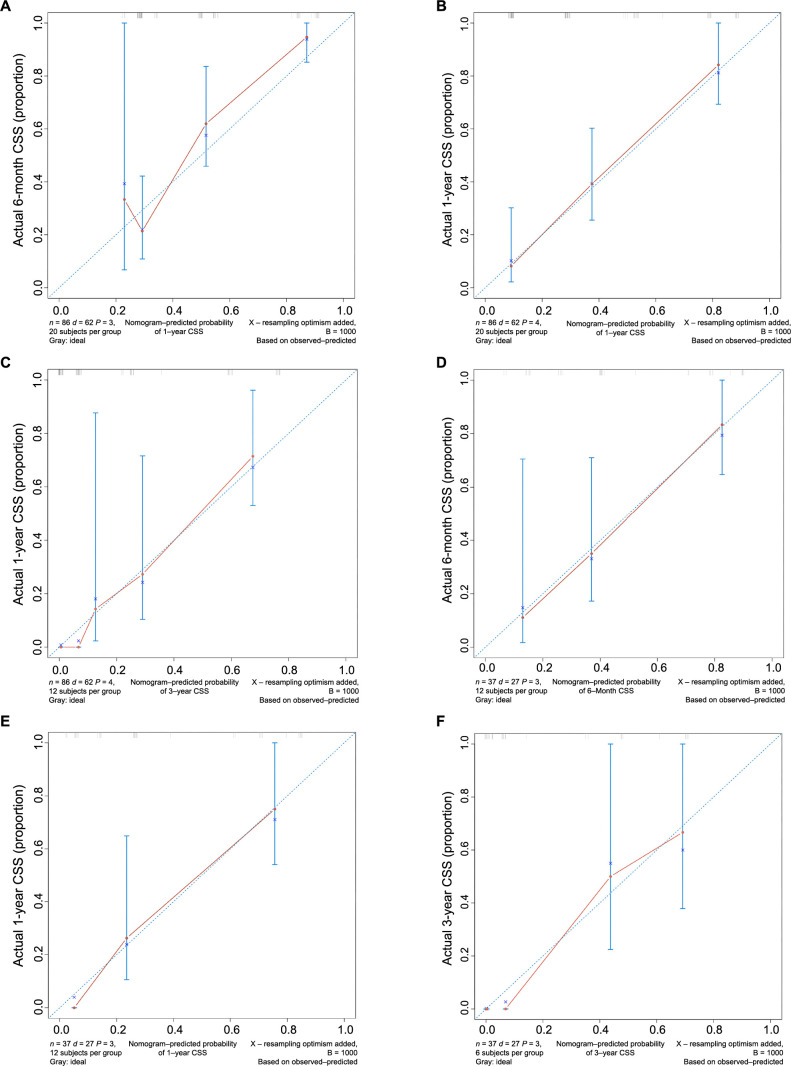
**The calibration plots between the nomogram and the actual observation in the training (A–C) and validation (D–F) cohorts for predicting the probability of 6-month, 1-, and 3-year CSS.** The x-axis represents the survival rate of individuals predicted by the model, and the y-axis represents the actual survival of individuals. CSS: Cancer-specific survival.

**Figure 6.**
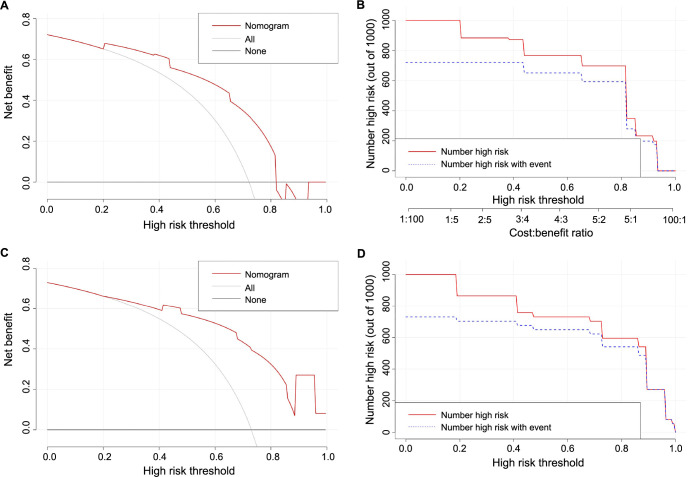
**The decision curves (A and C) and clinical impact curves (B and D) for predicting patient survival in the training (A and B) and validation (C and D) cohorts.** In the decision curves, the x-axis represents the survival rate of individuals predicted by the model, and the y-axis represents the actual survival of individuals. In the clinical impact curves, the red curve (high-risk count) represents the number of people classified as positive (high risk) by the nomogram model under each threshold probability, the blue curve (high-risk count with events) is the number of patients with actual events under each threshold probability.

**Figure 7.**
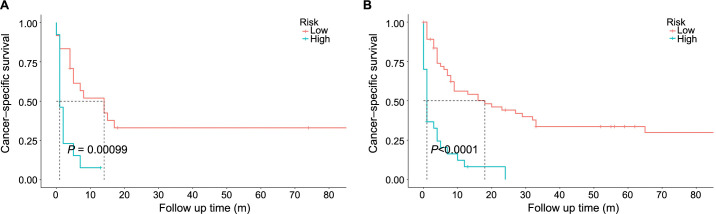
**The Kaplan–Meier CSS curves for low- and high-risk patients in the training (A) and validation cohorts (B).** CSS: Cancer-specific survival.

Based on the results from the multivariate analysis, M stage, surgery, and chemotherapy were included in the nomogram (Figure 3). Total points could be added up to calculate the 6-month, 1-year, and 3-year CSS for patients. Based on the clinical characteristics, the risk score was calculated for each patient. A higher score represented a lower survival possibility. The C-indices of the training cohort and validation cohort were 0.758 and 0.745, respectively. The ROC curves presented with AUC values are shown in (Figure 4). The training cohorts of the 6-month, 1-, and 3-year CSS had AUC values of 0.811 (95% CI 0.65–0.95), 0.830 (95% CI 0.66–0.98), and 0.914 (95% CI 0.58–0.99), respectively. The validation group, which had AUC values of 0.832 (95% CI 0.70–0.89), 0.872 (95% CI 0.71–0.92), and 0.888 (95% CI 0.82–0.98), respectively, demonstrated a similarly good discriminating ability. By plotting the calibration curve, the observed results were highly consistent with the predicted results in both training and validation groups (Figure 5). The DCA curves and clinical impact curves of both groups further demonstrated our model could be effective in clinical practice with considerable net clinical benefits (Figure 6). Furthermore, this nomogram’s C-index (0.758) was greater than the AJCC staging system’s 6th edition (0.728), suggesting the model’s quite good predictive ability. According to the survival analysis, patients with high-risk scores presented with poorer CSS compared with patients with low-risk scores (Figure 7).

### Dynamic nomogram

**Figure 8.**
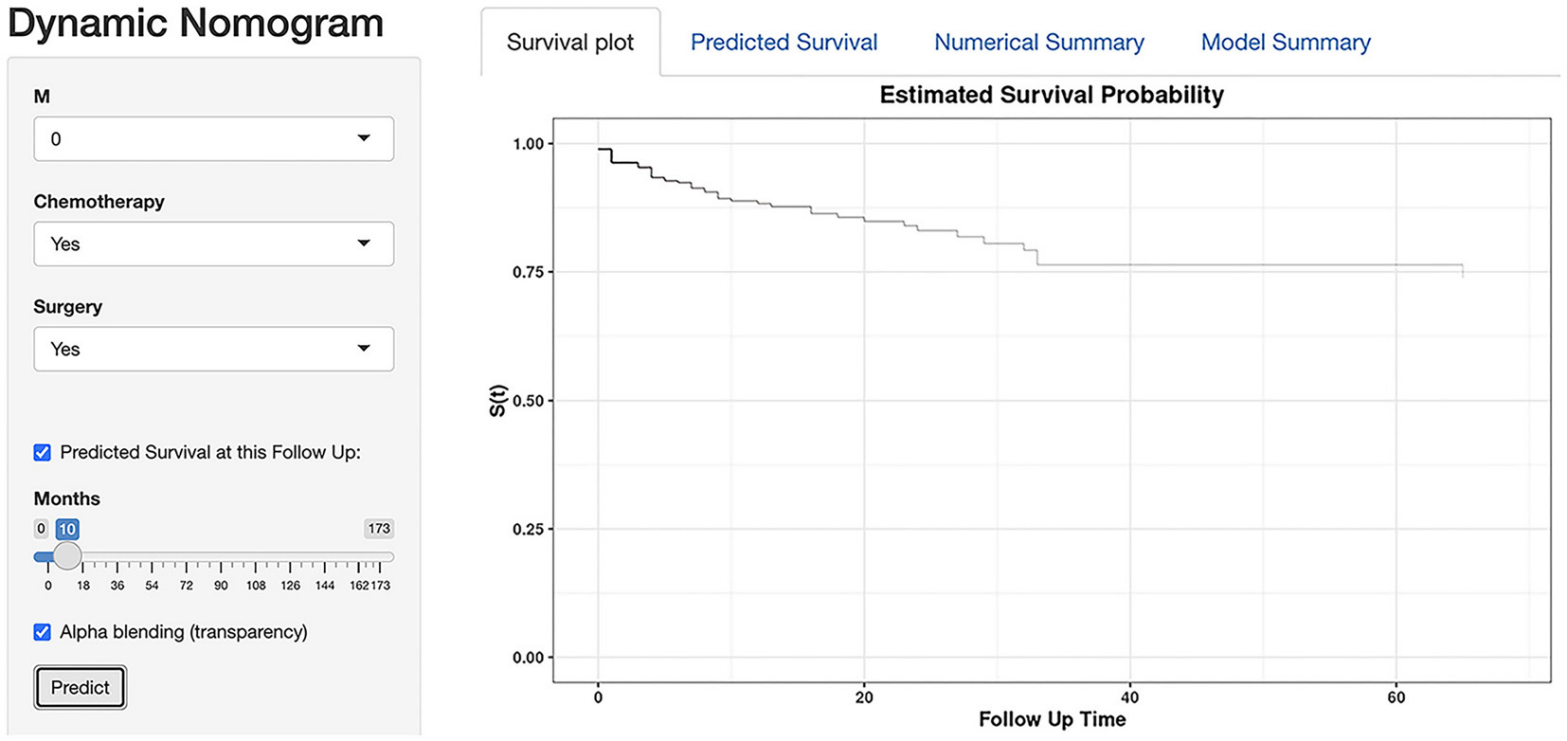
**A sketch map of dynamic nomogram for HAC**. Available at https://april-1998.shinyapps.io/dynamic_nomogram/. HAC: Hepatoid adenocarcinoma.

We utilized R software to create a dynamic nomogram. This dynamic model is accessible at https://april-1998.shinyapps.io/dynamic/_nomogram/. The predicted survival rate is displayed on the right side of the screen after each parameter is set on the left side of the sketch map (Figure 8).

## Discussion

HAC is a very rare and specific type of adenocarcinoma that originates outside the liver and has morphological and immunohistochemical characteristics similar to HCC. Initially, Ishikura et al. [11] proposed the concept of HAC because they observed high expression of AFP in some patients with gastric adenocarcinoma. HAC can originate in different organs, with the stomach and lung being the largest sites of origin. It is more common in elderly patients, with strong aggressiveness, low survival rate, and often early metastasis to the liver and lymph nodes, and the prognosis is far worse than that of ordinary adenocarcinoma [12–14]. Currently, timely detection and complete surgical resection are considered vital treatment strategies. Accurate differentiation between HCC and liver metastases of HAC is essential [8]. Due to the low incidence of HAC, few domestic reports, and the specificity of the clinical and imaging features of patients is not high, it is not easy to predict the prognosis. Our study constructed a novel nomogram to predict the prognosis of HAC patients based on the existing clinicopathological parameters.

The most common sites were previously reported as lymph nodes (57.5%) and liver (46.3%), followed by the lungs (3.4%) [15], compared with 54.5%, 16.2%, and 12.2% of our study, respectively. Our univariate and multivariate Cox regression analysis revealed that M stage, surgery, and chemotherapy were determined as independent prognostic factors, which are generally consistent with previous research [16]. Research utilizing the SEER database has shown that survival is better in patients under 60 years old, with no distant metastases, and who received surgery and treatment [17]. Our research, however, failed to find any independent correlations between age and CSS (Univariate Cox: *P* ═ 0.078). Distant metastases are more common in patients with HAC compared to other adenocarcinomas and are associated with a worse prognosis [15]. Our study revealed that the M1 stage is an independent poor prognostic factor of CSS in HAC patients (HR ═ 2.368, *P* ═ 0.039). Regarding treatments, surgery is recommended as the primary option for HAC at various sites and has been shown to prolong OS, particularly with radical surgery [18]. However, a previous study of HAC at the lung (HAL) reported no conventional treatments, including surgery, chemotherapy, and radiotherapy, could have survival benefits in patients with HAL (*P* > 0.05) [19]. On the contrary, our study demonstrated the promising efficacy of patients with HAC receiving surgeries (HR ═ 0.251, *P* ═ 0.008). The standard chemotherapy regimen of HAC remains to be settled, yet a case series of eight patients treated with fluoropyrimidine, platinum, paclitaxel, and cisplatin, indicated that HAC might be correlated with poor sensitivity to chemotherapy with a response rate of 8% and a disease control rate of 50%, implying the possible high resistance of HAC to chemotherapy [20, 21]. Controversially, cisplatin-based chemotherapy is considered the most effective first-line systemic treatment for metastatic HAC, with a promising clinical response observed in 75% of patients [22]. Our study also demonstrated that receiving chemotherapy is correlated with better CSS among patients with HAC (HR ═ 0.505, *P* ═ 0.024). Therefore, the efficacy of chemotherapy requires further research.

A nomogram based on 315 individuals with primary HAS revealed that node category 3b, CEA levels of 5 ng/mL or higher, and perineural invasion were all independent risk factors for poorer survival outcomes [23]. However, this study did not find any independent prognostic factors that are similar to our research. That nomogram based on these factors achieved C-indexes of 0.72 in the training cohort and 0.72 in the validation cohort, compared with 0.758 and 0.745 of our nomograms. Besides the encouraging C-indices, our AUC values also demonstrated good discriminant capacity in the validation group, which attained AUC values of 0.832, 0.872, and 0.888 for 6-month, 1-, and 3-year CSS, respectively. Another nomogram study of HAC indicated that age, preoperative CEA, number of examined lymph nodes, perineural invasion, and proportion of positive lymph node, have prognostic value on recurrence-free survival (RFS) (C-index 0.723) [9]. The AUC values of that study for 1-, 2- and 3-year RFS prediction were 0.741, 0.757, and 0.761, respectively, while our AUC values for 6-month, 1-, and 3-year CSS prediction were 0.80, 0.82, and 0.88, respectively. Generally, our research has shown considerable efficiency in predicting CSS among patients with HAC and firstly established a dynamic nomogram of HAC.

There are limitations to this study. First, there will inevitably be internal biases and limited significance because of the retrospective nature and absence of randomization. Second, access to comprehensive information on available treatments, molecular type, tumor markers, such as AFP and CEA, and other relevant data is unattainable due to restricted variables in the SEER database. These indicators are required for a thorough prognosis evaluation. The results could be impacted by differences in treatment philosophy between the SEER and validation sets. Because external validation was hampered by the low prevalence of HAC, statistical power for subgroup analysis was constrained. Integrating HAC patient data from many sources could be beneficial for future studies. Moreover, the follow-up period in our study is too short; extending it could increase the accuracy of the predictive model.

## Conclusion

Based on three variables (M stage, surgery, and chemotherapy) that correlate significantly with CSS, we generated specific nomograms to improve the long-term prognosis prediction of patients with HAC. These nomograms can lead to more targeted treatment and follow-up strategies for HAC. To our knowledge, this is the first time nomograms have been used to predict CSS in HAC patients. The nomogram showed good performance in both the training and validation groups. Furthermore, the nomogram effectively differentiates between high- and low-risk patients, revealing a significant difference in survival rates between the two groups. Our study’s nomogram can be a useful tool for predicting HAC prognosis, regardless of location, as it can divide patients into high- and low-risk categories.

## Data Availability

The datasets analyzed during the current study are available in the SEER repository (https://seer.cancer.gov/data-software/). The dynamic nomogram is available at https://april-1998.shinyapps.io/dynamic_nomogram/.

## References

[1] Yang J, Wang R, Zhang W, Zhuang W, Wang M, Tang C (2014). Clinicopathological and prognostic characteristics of hepatoid adenocarcinoma of the stomach. Gastroenterol Res Pract.

[2] Inoue M, Sano T, Kuchiba A, Taniguchi H, Fukagawa T, Katai H (2010). Long-term results of gastrectomy for alpha-fetoprotein-producing gastric cancer. Br J Surg.

[3] Arnould L, Drouot F, Fargeot P, Bernard A, Foucher P, Collin F (1997). Hepatoid adenocarcinoma of the lung: report of a case of an unusual alpha-fetoprotein-producing lung tumor. Am J Surg Pathol.

[4] Burgués O, Ferrer J, Navarro S, Ramos D, Botella E, Llombart-Bosch A (1999). Hepatoid adenocarcinoma of the urinary bladder. An unusual neoplasm. Virchows Arch.

[5] Aizawa K, Motoyama T, Suzuki S, Tanaka N, Yabusaki H, Tanaka S (1994). Different characteristics of hepatoid and non-hepatoid alpha-fetoprotein-producing gastric carcinomas: an experimental study using xenografted tumors. Int J Cancer.

[6] Devouassoux-Shisheboran M, Schammel DP, Tavassoli FA (1999). Ovarian hepatoid yolk sac tumours: morphological, immunohistochemical and ultrastructural features. Histopathology.

[7] Ishikura H, Kishimoto T, Andachi H, Kakuta Y, Yoshiki T (1997). Gastrointestinal hepatoid adenocarcinoma: venous permeation and mimicry of hepatocellular carcinoma, a report of four cases. Histopathology.

[8] Huang ZN, Huang YQ, Hong QQ, Zhang P, Zhang ZZ, He L (2023). Long-term prognostic benefit of adjuvant chemotherapy for patients with hepatoid adenocarcinoma of the stomach after radical resection: a national multicenter study. Eur J Surg Oncol.

[9] Lin JX, Lin JP, Hong QQ, Zhang P, Zhang ZZ, He L (2023). Nomogram to predict recurrence and guide a pragmatic surveillance strategy after resection of hepatoid adenocarcinoma of the stomach: A retrospective multicenter study. Ann Surg Oncol.

[10] Simmet V, Noblecourt M, Lizée T, Morvant B, Girault S, Soulié P (2018). Chemotherapy of metastatic hepatoid adenocarcinoma: Literature review and two case reports with cisplatin etoposide. Oncol Lett.

[11] Ishikura H, Kanda M, Ito M, Nosaka K, Mizuno K (1990). Hepatoid adenocarcinoma: a distinctive histological subtype of alpha-fetoprotein-producing lung carcinoma. Virchows Archiv A Pathol Anat.

[12] Xia R, Zhou Y, Wang Y, Yuan J, Ma X (2021). Hepatoid adenocarcinoma of the stomach: current perspectives and new developments. Front Oncol.

[13] Li M, Fan Y, Lu H (2021). Hepatoid adenocarcinoma of the lung. Technol Cancer Res Treat.

[14] Zhou K, Wang A, Ao S, Chen J, Ji K, He Q (2020). The prognosis of hepatoid adenocarcinoma of the stomach: a propensity score-based analysis. BMC Cancer.

[15] Su JS, Chen YT, Wang RC, Wu CY, Lee SW, Lee TY (2013). Clinicopathological characteristics in the differential diagnosis of hepatoid adenocarcinoma: a literature review. World J Gastroenterol.

[16] Zeng XY, Yin YP, Xiao H, Zhang P, He J, Liu WZ (2018). Clinicopathological characteristics and prognosis of hepatoid adenocarcinoma of the stomach: Evaluation of a pooled case series. Current Med Sci.

[17] Wang W, Li G (2020). Incidence and prognostic factors of hepatoid adenocarcinoma: a population-based analysis. Transl Cancer Res.

[18] Kang-Tao Wang, Meng-Xiang Tian, He-Ming Ge, MaiMaiti Aizezi, Fang Song, JIng Deng (2022). Meta-analysis of 294 cases of gastrohepatoid adenocarcinoma in China. China J Gen Surg.

[19] Lei L, Yang L, Xu YY, Chen HF, Zhan P, Wang WX (2021). Hepatoid adenocarcinoma of the lung: an analysis of the Surveillance, Epidemiology, and End Results (SEER) database. Open Med (Warsaw, Poland).

[20] Baek SK, Han SW, Oh DY, Im SA, Kim TY, Bang YJ (2011). Clinicopathologic characteristics and treatment outcomes of hepatoid adenocarcinoma of the stomach, a rare but unique subtype of gastric cancer. BMC Gastroenterol.

[21] Liu Z, Wang A, Pu Y, Li Z, Xue R, Zhang C (2021). Genomic and transcriptomic profiling of hepatoid adenocarcinoma of the stomach. Oncogene.

[22] Simmet V, Noblecourt M, Lizée T, Morvant B, Girault S, Soulié P (2018). Chemotherapy of metastatic hepatoid adenocarcinoma: Literature review and two case reports with cisplatin etoposide. Oncol Lett.

[23] Lin JX, Wang ZK, Hong QQ, Zhang P, Zhang ZZ, He L (2021). Assessment of Clinicopathological characteristics and development of an individualized prognostic model for patients with hepatoid adenocarcinoma of the stomach. JAMA Netw Open.

